# GRE T2^∗^-Weighted MRI: Principles and Clinical Applications

**DOI:** 10.1155/2014/312142

**Published:** 2014-04-16

**Authors:** Meng Yue Tang, Tian Wu Chen, Xiao Ming Zhang, Xiao Hua Huang

**Affiliations:** Sichuan Key Laboratory of Medical Imaging, Department of Radiology, Affiliated Hospital of North Sichuan Medical College, Nanchong, Sichuan 637000, China

## Abstract

The sequence of a multiecho gradient recalled echo (GRE) T2*-weighted imaging (T2*WI) is a relatively new magnetic resonance imaging (MRI) technique. In contrast to T2 relaxation, which acquires a spin echo signal, T2* relaxation acquires a gradient echo signal. The sequence of a GRE T2*WI requires high uniformity of the magnetic field. GRE T2*WI can detect the smallest changes in uniformity in the magnetic field and can improve the rate of small lesion detection. In addition, the T2* value can indirectly reflect changes in tissue biochemical components. Moreover, it can be used for the early diagnosis and quantitative diagnosis of some diseases. This paper reviews the principles and clinical applications as well as the advantages and disadvantages of GRE T2*WI.

## 1. Introduction

The sequence of a multiecho gradient recalled echo (GRE) T2*-weighted imaging (T2*WI) acquires a gradient echo signal, and depending on the technical characteristics, there are some advantages of this technique. On the one hand, the sequence of GRE T2*WI requires high uniformity of the magnetic field, independent of the paramagnetic substance or diamagnetic substance. With changes in the uniformity of the magnetic field, the sequence is sensitive for detection and can improve the detection rate of small lesions [1]. On the other hand, different tissues and organs have different T2* values, and the different statuses of the same tissues and organs also have different T2* values. Thus, the T2* value has the potential to reflect a change in the biochemical components of the counterpart and may be used for the early diagnosis and quantitative diagnosis of some diseases [2]. Currently, this technology has been reported in clinical applications, particularly in the study of cerebral microbleeds [3, 4] and iron deposition [5]. This paper reviews the principles and clinical applications as well as advantages and disadvantages of GRE T2*WI.

## 2. Principles of GRE T2*WI [4, 6]

The principles of multiple echo GRE T2*WI sequence are similar to that of conventional T2-weighted imaging (T2WI) [2]. T2 relaxation of T2WI uses the 180° refocusing pulse to eliminate the inhomogeneity of the main magnetic field after turning off the RF (radio frequency). T2 relaxation only reflects the decay of the transverse magnetization vector of the tissue itself. However, T2* relaxation does not use the 180° refocusing pulse to focus the pulses but uses it to switch the gradient field to generate the signal reunion after turning off the RF. T2* relaxation reflects the decay of the transverse magnetization vector, which is caused by the combination of two factors; the first factor is the tissue itself, and the second factor is the inhomogeneity of the main magnetic field.

The T2* value is a quantifiable indicator of GRE T2*WI that is related to the T2 value. It is presented in the following equation, which can be expressed as the interrelationship between T2 and T2* [7]:

(1) $$\begin{array}{c} \frac{1}{\text{T}2^{*}}=\frac{1}{\text{T}2}+\gamma \cdot \Delta B_{\text{inhom}}. \end{array}$$

*Note. γ* represents the magnetogyric ratio and Δ*B*_inhom_ represents the magnitude of the magnetic field changes. Assuming that the applied static magnetic field is uniform (*B*0 = 0), then the T2* term is only affected by the “γ*Δ*B*_inhom_,” which is influenced only by local susceptibility fields. Thus, the T2* term is affected by the local susceptibility fields. This is the theoretical basis by which the GRE T2*WI can detect the smallest changes in uniformity in a magnetic field [2].

Compared with T2 relaxation, which is based on the principle of the sequence of GRE T2*WI, the decay of T2* relaxation was added as a factor of the main magnetic field. On the one hand, for the same tissue, the decay time of T2* relaxation is shorter compared to the T2 relaxation. On the other hand, the sequence of GRE T2*WI requires high uniformity of the magnetic field. GRE T2*WI can detect the smallest changes in uniformity in the magnetic field and can improve the detection rate of a small lesion, such as the paramagnetic substance deposition, similar to iron deposition [8].

T2* relaxation acquires the gradient echo signal. First, it acquires a series of T2* images using the same TR scan time and a series of TE times. Subsequently, the T2* value of each pixel was calculated by selecting the appropriate regions of interest (ROI). Finally, T2* relaxation constitutes the color gradation, which can be quantitatively analyzed, and it is also called the T2* relaxation time figure or T2* map. This process is completed using postprocessing software directly after scanning.

## 3. Clinical Application

### 3.1. Imaging Assessment of Small Lesions, Such as Iron Deposits and Microbleeds

#### 3.1.1. GRE T2*WI for Iron Deposition

Iron is present throughout the human body in tissue cells and is indispensable to human survival. However, the abnormal deposition of iron can disrupt the balance of iron homeostasis in a number of tissues. Iron deposition is common in clinical diseases, such as chronic liver disease [9, 10] and chronic anemia [11]. Because iron is paramagnetic, when iron deposition increases, the uniformity of the local magnetic field changes to various degrees depending on the content of the iron deposition. Thus, iron deposition can result in an increase in susceptibility effects, thereby affecting the T2* relaxation time [11]. One of the advantages of GRE T2*WI is to use different TE times to acquire a series of T2* images. These images can be used to quantitatively measure the T2* values of the tissues using postprocessing software.

**Figure 1:**
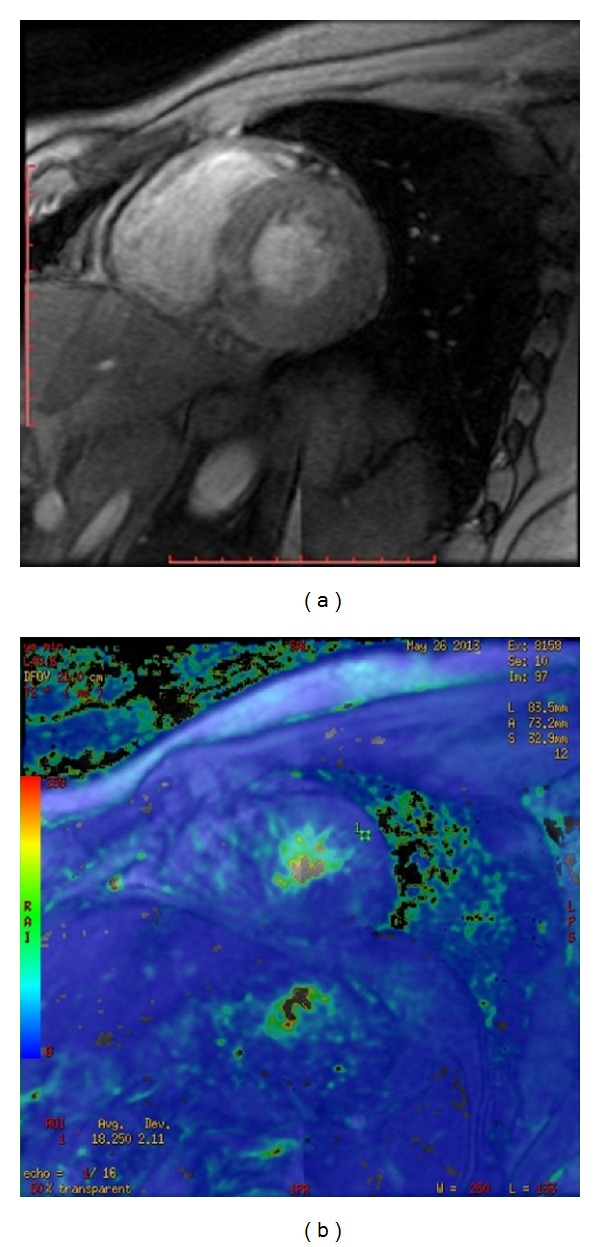
A 25-year-old young healthy male volunteer. GRE T2*WI reveals the ventricular central T2* figure (a) and color map (b) on the left ventricular short axis dimension.

**Figure 2:**
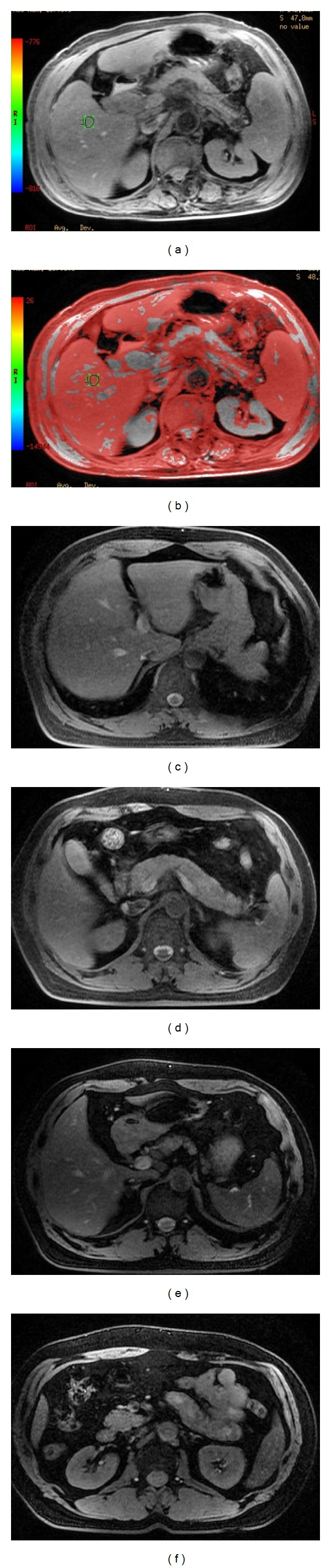
A 59-year-old man with mild anemia. The T2* (a) and corresponding T2* color maps (b) of the upper abdomen were derived from T2*WI. The liver, spleen, and pancreas can be visualized by GRE T2*WI (c–f). The T2* values of the tissues and organs can also be measured using the same sequence.

**Figure 3:**
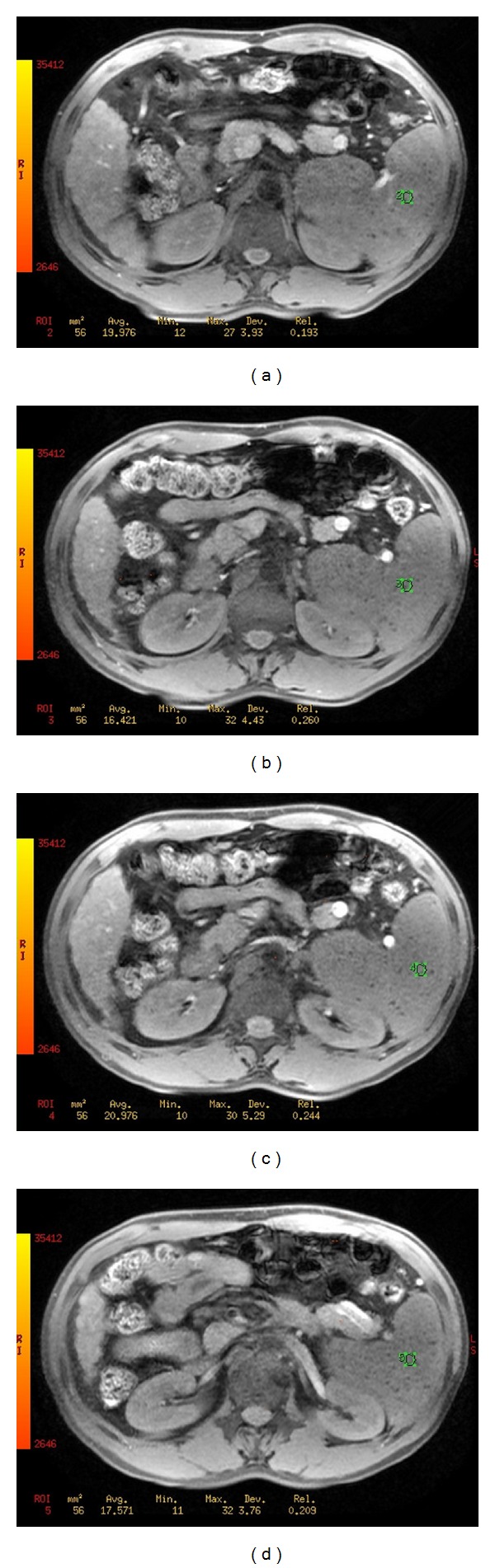
A 62-year-old man with liver cirrhosis and hypersplenotrophy. The liver and spleen can be visualized by GRE T2*WI (a–d). The T2* values of the spleen can be quantitatively measured and can be used to evaluate the iron overload of the spleen.

As a quantitative indicator, the T2* value is widely used in clinical applications. In addition, the measurement of the T2* value of tissues on the GRE T2*WI can indirectly reflect the iron content of the tissues, which demonstrates two advantages: noninvasiveness and rapidness. The applied MRI GRE T2*WI technique has been previously validated for the quantitative determination of changes in tissue biochemical components [12]. The increase in iron content of tissues can cause a shortening in the T2* relaxation time. This is conducive to quantitatively study iron deposition. Several previous studies on GRE T2*WI for iron deposition in many organs [9, 10, 13–15] (Figures 1, 2, and 3), particularly in the liver, heart, and brain, have been performed.

Although the determination of the liver iron concentration (LIC) is the gold standard to evaluate the body iron load conditions via liver biopsies, this examination is invasive and is limited by its sample size and thus is not conducive for the expansion of clinical application. Queiroz-Andrade et al. [16] studied the MRI findings of iron overload and demonstrated the use of GRE T2*WI as a sensitive tool to detect iron overload. In 2013, Henninger et al. [9] used GRE T2*WI to evaluate liver fat in the presence of iron. Their results showed that GRE T2*WI helped to quantify the iron content and was conducive in diagnosing hepatic fat in the presence of hepatic iron overload (HIO).

Chronic anemia can cause iron deposition in the heart. In 2012, Barzin et al. [11] reported a correlation of cardiac MRI T2* with echocardiography in the thalassemia major and showed that the T2* values decrease (<20 ms; the mean value of cardiac T2*: 20.41 ± 12.1 ms) in patients with thalassemia.

GRE T2*WI can also evaluate the iron deposition caused by some nervous system diseases, such as brain iron deposition in Alzheimer's disease. Qin et al. [17] quantitatively determined the specific area of interest of the T2* value and performed a correlation analysis between the T2* value and iron in the body load. They found that Alzheimer's disease classification and iron load state were highly correlated and consistent with the results of biochemical analyses. McNeill et al. [18] used the same methods to perform a correlation study between iron deposits and neural degeneration and showed that T2*WI could distinguish between the different subtypes of neurodegeneration associated with brain iron accumulation.

Mihai et al. [19] studied atherosclerosis in patients at high risk of atheromatous plaques of the carotid artery blood wall using GRE T2*WI and confirmed that the iron load was correlated with atheromatous plaque formation. Their study indicated that, to a specific extent, the state of high iron load could inhibit the formation of atheromatous plaques.

GRE T2*WI may also detect disease progression and may be combined with other related indicators to evaluate the changes in the organ functional state, such as the T2* value, which can be combined with P-wave changes to assess the myocardial iron load status [20]. In some related animal studies, it was reported that the GRE T2*WI technique is also a good tool to sensitively detect paramagnetic material, such as ultrasmall superparamagnetic particles of iron oxides (USPIO). Its appearance on the GRE T2*WI is a local signal loss [21, 22].

#### 3.1.2. Detection and Evaluation of Microbleeds [23]

**Figure 4:**
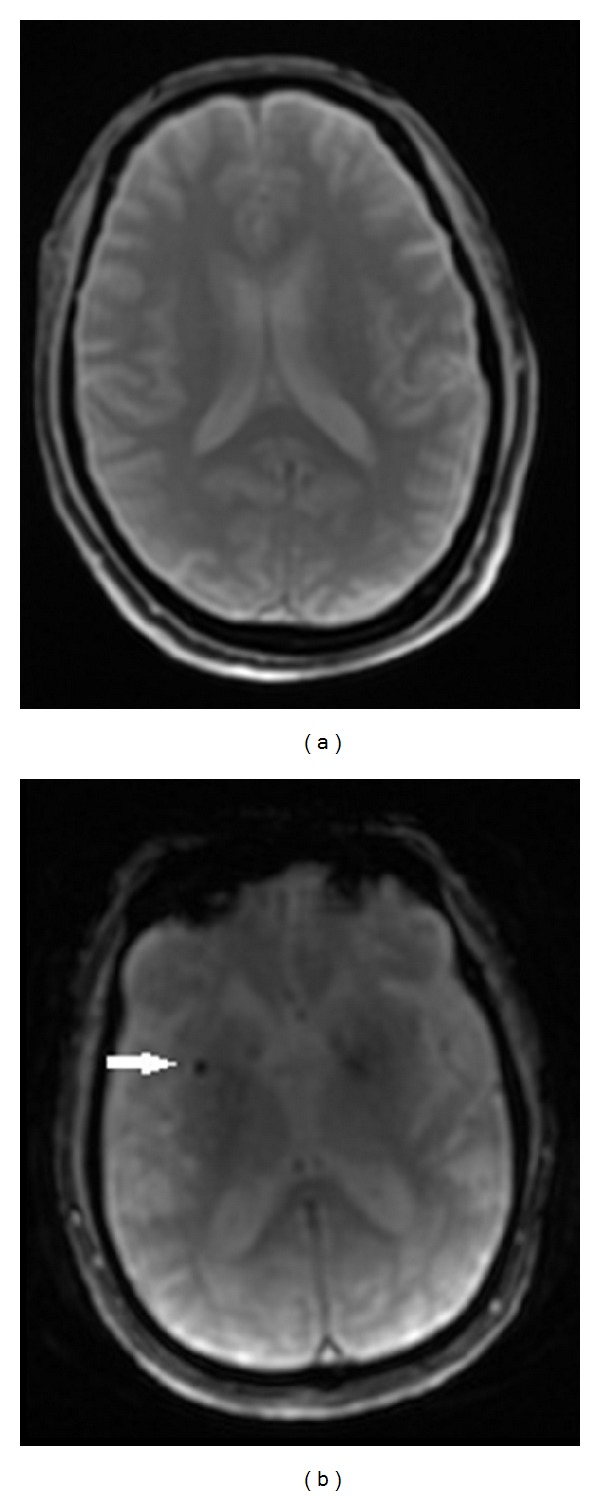
A 60-year-old healthy male volunteer. The T2* (a) shows a section of the basal ganglia. A 79-year-old man who experienced dizziness. GRE T2*WI (b) demonstrated cerebral microbleeds, which appeared as small, rounded, homogeneous, dark dot-like lesions (arrow).

Cerebral microbleeds (CMBs) are commonly and frequently encountered in small vessel diseases among elderly people with hypertensive arteriopathy and cerebral amyloid angiopathy [24]. CMBs are defined as a radiological entity; they are small, rounded, homogeneous, hypointense lesions on GRE T2*WI [25]. The CBM lesions cannot be detected on the conventional spin-echo MRI sequences, but GRE T2*WI can be visually confirmed to identify microbleeds [26] (Figure 4).

The intracranial manifestation of blood on an MRI is complicated and is dependent on its evolution over time and its location [27–30]. Oxygen inside the red blood cells, the content of oxygenated hemoglobin (oxyHb), and the integrity of red blood cells determine the level of paramagnetic material effect in blood. When the bleeding breakdown products go through several stages of evolution over time, the uniformity of microscopic local magnetic fields changes. The early evolution is from oxyhemoglobin to deoxyhemoglobin. The deoxyhemoglobin has paramagnetic effects, and the magnetic susceptibility effects can result in a signal loss (darkening), such that it can be sensitively detected on the GRE T2*WI. Bleeding breakdown products have a large paramagnetic effect or recalled magnetic susceptibility effects, which can result in a signal loss (darkening) in specific deoxygenated hemoglobin (deoxyHb), intracellular methemoglobin (MetHb), and hemosiderin contained within perivascular macrophages [31, 32]. Thus, it can result in small changes in the microscopic local magnetic fields and shortening of the T2* relaxation time. In addition, the GRE T2*WI sequence can be applied to detect a small hemorrhage lesion caused by the local field inhomogeneities [6], and it is very sensitive.

On the basis of imaging characteristics, the GRE T2*WI can diagnose microbleeds early, which is more conducive for clinical treatment. In 1996, Patel et al. [33] studied the hyperacute primary intraparenchymal hemorrhage using MRI, and their results indicated that, for hyperacute primary intraparenchymal hemorrhage (within the first few hours), GRE T2*WI can detect a hemorrhage lesion. In addition, Kaya et al. [34] studied acute ischemic infarction using GRE T2*WI with a 3.0T MR scanner and found that GRE T2*WI can be used as a supportive imaging technique for the diagnosis of hyperacute ischemic stroke. Moreover, regions of marked signal loss on GRE T2*WI images [35] can be used for the purpose of localizing diagnosis early and to provide evidence for early clinical treatment.

GRE T2*WI has a higher detection rate than conventional MRI sequences and CT examinations for cerebral hemorrhages, taking advantage of its ability to detect small bleeding lesions [36]. Compared with a conventional sequence, the GRE T2*WI sequence not only improves the detection rate of microbleeds but also reveals the progression of the bleeding lesion. Because the GRE T2*WI is sensitive to paramagnetic materials, it can identify more lesions that contain paramagnetic matter and can reveal details of the lesions [37, 38]. Thus, it shows a more accurate volume of the hematoma during its progression. Furthermore, it was pathologically confirmed that the scope of iron deposition corresponds with the scope of the revealed lesions on the GRE T2*WI [33].

#### 3.1.3. Related Studies on Degenerative Diseases

The intervertebral disc mainly consists of the nucleus pulpous, annulus fibrous, and cartilage endplate. Its degeneration is affected by robust factors and physiological degeneration. Degeneration can result in altered biochemistry, thereby affecting the mechanical competence [39, 40]. The main feature of the early degeneration of the intervertebral disc tissue is changes in the biochemical composition of the nucleus pulpous, which includes decreases in protein polysaccharide concentration, low moisture content, and alterations in collagen elements [41]. Degeneration of the intervertebral disc occurs earlier and is more obvious compared to the annulus fibrous and cartilage endplate [42]. Although conventional MRI sequences can clearly reveal the morphological features of the intervertebral disc, early degeneration of the intervertebral disc can be visualized with changes in the nucleus pulpous biochemical composition and thus clear images are limited and may not be adequate in detecting early changes in degeneration [43].

**Figure 5:**
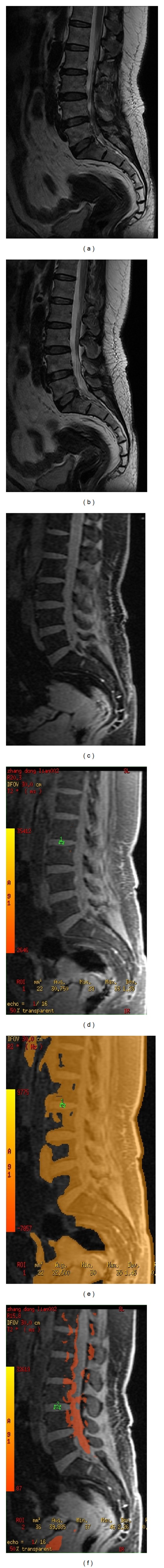
A 53-year-old female without any back pain symptoms. The T2 (a-b) and T2* (c), corresponding to the T2* measurement drawing (d–f), and color maps (e-f) of intervertebral discs were derived from T2*WI. The intervertebral discs are visualized using T2WI and GRE T2*WI with no obvious degeneration. The T2* values of the corresponding intervertebral discs can also be quantitatively measured and can detect early degeneration.

**Figure 6:**
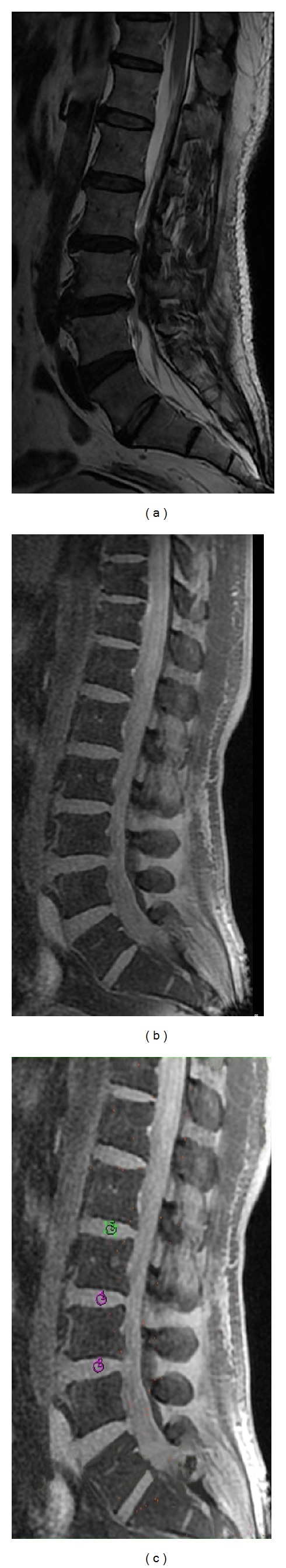
A 66-year-old man with Lumbosacral pain. T2WI (a) clearly reveals the degenerative changes of lumbar spine. The T2* and corresponding T2* measurement drawing (b-c) were derived from T2*WI. The T2* values of the intervertebral discs can also be quantitatively measured to quantify the degeneration.

GRE T2*WI can provide a new examination method for the early diagnosis of degenerative diseases caused by changes in the biochemical composition and lack of functional changes in early disease [44]. It provides the quantitative T2* value and is also dominant in the display of small structures [44, 45]. In addition, reduced protein polysaccharide concentrations and an increase in free water are observed [41] in processes of early degeneration, which results in an increase in the T2* value. Perry et al. [43] and Takashima et al. [41] studied the value of T2 measurements as a measure of disk degeneration. Their results indicated that the indicators of T2 values can quantitatively evaluate early degeneration. Welsch et al. [46] reported that the GRE T2*WI not only quantitatively measures the T2* value of the intervertebral disc nucleus pulpous but also diagnoses early degeneration. Thus, measurement of the T2* value on T2*WI (Figures 5 and 6) may provide new evidence for the early clinical treatment of some patients with early intervertebral disc degeneration.

Osteoarthritis is common articular cartilage degeneration that is accompanied by changes in the chronic degeneration of the subchondral bone joint and surrounding tissue. This type of degenerative change is most common in the knee joint. In addition, early changes in lesions include changes in the collagen fiber [47], which results in an increase in water permeability and causes the outflow of substrate moisture. Finally, it results in the relative increase of water content in the articular cartilage substrate [12, 48] and thus the T2* relaxation time will be extended. Several studies have shown that GRE T2*WI can be used to evaluate changes in the early articular cartilage degeneration of biological tissue structure with no obvious morphological changes [12, 49]. This has important clinical value for early articular cartilage degeneration. GRE T2*WI not only clearly shows the thin layer of cartilage but also quantitatively assesses the classification of the degeneration. In addition, it has a strong advantage in the functional study of joints [50].

#### 3.1.4. GRE T2*WI for Microdamage

Microdamage in the bone and joint is accumulated due to high-intensity training or normal daily activity [51, 52]. The burden of microdamage of bone in vivo is determined by the balance of the generation and repair of microdamage [53, 54]. GRE T2*WI can clearly show the articular cartilage and its surrounding tissue [12]. It is sensitive to changes in the minimal change of joints and even biochemical changes in the absence of morphological changes [12]. Microcontusion in the absence of morphological changes in the bone and joint can only be visualized in the local tissue edema physiologically. The relative increase in water content is the basis of selection for the GRE T2*WI scan. The T2* value increases to some extent [12], and the T2* value is a clinically feasible parameter for biochemical evaluation.

GRE T2*WI is able to detect microdamage that causes changes or imbalances in the tissue biochemical composition. It has a high spatial resolution image [2] for microdamage and is useful in visualizing small anatomical structures with conspicuity in the bone or joint [2] and in the ligament or meniscus [55, 56]. When a meniscal tear occurs, T2* mapping can be sensitive in detecting the meniscal structure and composition [57]. Not only can a clear T2* image be acquired but also a quantitative T2* value is available for quantitative analyses. Stelzeneder et al. [58] performed some studies on the evaluation of repair tissue quality following arthroscopic autologous collagen-induced chondrogenesis (ACIC) via the measurement of the T2* relaxation time values of repair tissue. They found that the T2* value can be used as a feasible monitoring parameter of tissue (hyaline cartilage) repair.

### 3.2. GRE T2*WI for Tumors

GRE T2*WI can be applied in tumors based on its high spatial resolution and sensitivity in nonhomogeneous tissues in the magnetic field.

Regarding its high spatial resolution, T2*WI is conducive in revealing the tumor mass and its adjacent invasion, such as a spinal tumor. Spinal tumors always invade accessories of the vertebral body, and GRE T2*WI shows a strong advantage in the imaging of accessories, which are similar to small lesions. The tumor has three clinical types, osteolytic type, osteoblastic type, and mixed type, and different tumors have different compositions oftumor body based on its pathology. The T2* value of the tumor mass can be quantitatively measured on the GRE T2*WI sequence, which may identify the differentiation of the tumor and can detect the paramagnetic substance, similar to the same type of tumor with a hemorrhage or with iron deposition.

Regarding its sensitivity for nonhomogeneous tissues in the magnetic field, the T2*-weighted first-pass perfusion imaging (T2*-PWI) and blood oxygenation level dependent (BOLD) imaging are both based on the GRE T2*WI technique, which are referred to as functional magnetic resonance imaging (fMRI) [59]. As noninvasive methods, the GRE T2*WI technique can be broadly applied in clinical tumors.

**Figure 7:**
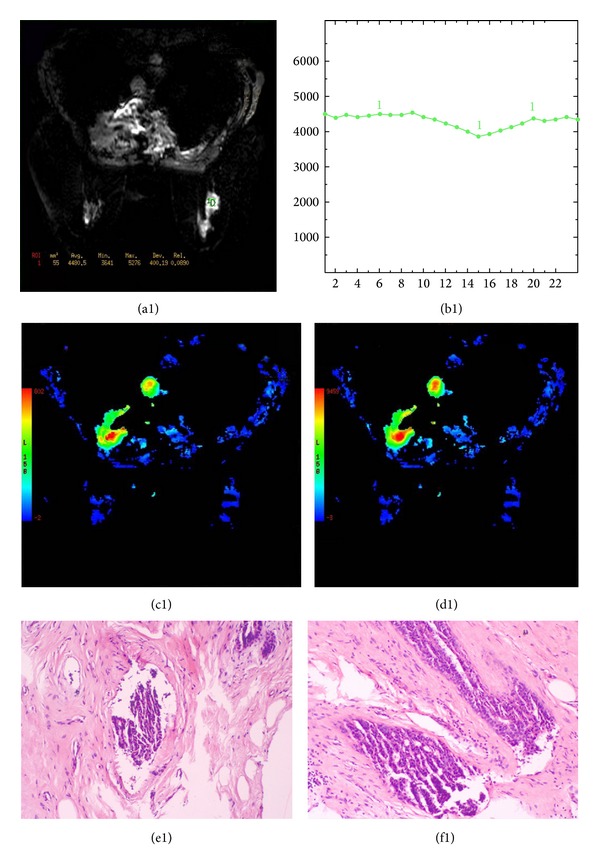
A 42-year-old female with a fibroadenoma of the right breast. T2*-PWI (a1) reveals a round and hyperintense lesion. The time to signal intensity curves (b1) and corresponding color maps (c1-d1) were derived from T2*-PWI. The maximum signal drop rate derived from postprocessing software is 16%. The lesion was confirmed pathologically (e1-f1).

**Figure 8:**
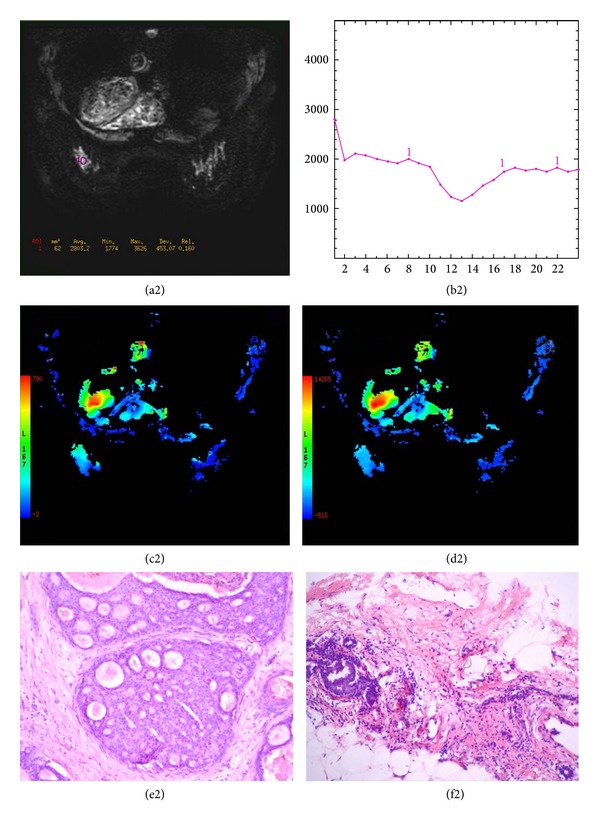
A 48-year-old female with an invasive ductal carcinoma of the left breast. The T2*-PWI (a2) reveals a round and hyperintense lesion. The time to signal intensity curves (b2) and corresponding color maps (c2-d2) were derived from T2*-PWI. The maximum signal drop rate derived from postprocessing software is 43.90%. The pathology of the lesion is shown in the ductal carcinoma (e2-f2).

T2*-PWI is widely used in breast tumors (Figures 7 and 8). Breast tumors have their own unique features in the first-pass perfusion images [60]. Because the GRE T2*WI is sensitive to the smallest changes in magnetic field, when the paramagnetic contrast agent (like Gd-DTPA) [61] reaches the microvessels of the tumor tissues for the first time, the GRE T2*WI can detect these changes sensitively, which appear as a decrease in signal intensity in T2*WI. The time to signal intensity curves were derived from postprocessing software via the selection of an appropriate region of interest on the T2*-weighted first-pass perfusion images (T2*-PWI). The point at the peak of the curves (the maximal signal intensity drop rate) can present the concentration of the contrast agent. In addition, T2*-PWI is conductive in evaluating the tumor microvascular distribution [62].

In the same respect, when the oxygenated hemoglobin is deoxygenated, it results in a significant magnetic field inhomogeneity in the vicinity [63]. GRE T2*WI can detect this change based on the local magnetic field inhomogeneity. To some extent, fMRI BOLD imaging can help to monitor tumor metabolism [64]. Similarly, GRE T2*WI can also be used to detect other tumors [65, 66].

## 4. Advantages and Disadvantages of GRE T2*WI

GRE T2*WI is a fast, convenient, noninvasive, and feasible technique that demonstrates obvious advantages in clinical applications. First, selecting shorter TR and TE can accelerate the pace of imaging and shorten the time of data collection. Second, in contrast with T2 relaxation, which acquires a spin echo signal, T2* relaxation acquires a gradient echo signal. Thus, in the echo formation process, without the phase reunion by the 180° focused pulse of SE sequence, GRE T2*WI is more sensitive to the nonuniformity of the magnetic field compared to conventional MRI scanning. Thus, the sequence of GRE T2*WI requires high uniformity of the magnetic field and can detect small changes in the uniformity in the magnetic field to improve the detection rate of small lesions [36]. Thus, it is easier to monitor inhomogeneous factors, such as bleeding and hemosiderin deposition, that can cause a nonuniformity of the local magnetic field [2]. Third, the precise quantifiable indicators and T2* relaxation time are more conducive for the quantitative evaluation of the physiological or pathological status of some tissues and organs.

However, currently, GRE T2*WI has some limitations that need to be improved in the future. Because the gradient echo sequence often uses the low-angle shot, the echo amplitude is often less than that of the SE sequence; thus, the GRE sequence image inherent signal-to-noise ratio is lower compared to the SE sequence. Therefore, it may be affected by the respiratory mobility in an abdomen MRI examination. In the postprocessing measurement, the results will be different due to the selection of regions of interest (ROI). In addition, the choice of parameters is important. For example, the TE echo time cannot be too long or too short. To properly extend the TE, we can achieve good T2* imaging, but an extension of TE must be under control. It causes a decrease in the signal intensity of the image and a more obvious deformation for unlimited extension. Thus, we should emphasize these related factors, which will affect the T2* value. Ultimately, the T2* value will be measured and will be available and convincing.

## 5. Summary

GRE T2*WI appears to be a promising MRI sequence for iron deposit and microbleeds. GRE T2*WI also facilitates the study on degenerative diseases, microdamage, and tumors. In this paper, we highlight the clinical applications of GRE T2*WI. As a noninvasive, reliable, sensitive MRI technique, GRE T2*WI not only improves the detection rate of small lesions but also provides physiological or pathological status indicators in the clinic for the quantitative evaluation of certain organs or tissues. GRE T2*WI is more useful for the diagnosis of a disease when combined with conventional MRI sequences, and GRE T2*WI will be widely used in clinical applications.
